# Little evidence for association between the TGFBR1*6A variant and colorectal cancer: a family-based association study on non-syndromic family members from Australia and Spain

**DOI:** 10.1186/1471-2407-14-475

**Published:** 2014-07-01

**Authors:** Jason P Ross, Linda J Lockett, Bruce Tabor, Ian W Saunders, Graeme P Young, Finlay Macrae, Ignacio Blanco, Gabriel Capella, Glenn S Brown, Trevor J Lockett, Garry N Hannan

**Affiliations:** 1 CSIRO Preventative Health Flagship, Sydney, NSW, Australia; 2 CSIRO Animal, Food and Health Sciences, Sydney, NSW, Australia; 3 CSIRO Computational Informatics, Sydney, NSW, Australia; 4 CSIRO Computational Informatics, Adelaide, SA, Australia; 5 Flinders Centre for Cancer Prevention and Control, Flinders University, Adelaide, SA, Australia; 6 Department of Medicine, University of Melbourne, and Colorectal Medicine and Genetics, The Royal Melbourne Hospital, Melbourne, VIC, Australia; 7 Cancer Genetic Counseling Program and Translational Research Laboratory, Institut Català d’Oncologia-IDIBELL and University of Barcelona, L’Hospitalet de Llobregat, 08907 Barcelona, Spain

**Keywords:** TGFBR1, 6*A, rs11466445, Colorectal, Cancer, Hereditary

## Abstract

**Background:**

Genome-wide linkage studies have identified the 9q22 chromosomal region as linked with colorectal cancer (CRC) predisposition. A candidate gene in this region is transforming growth factor β receptor 1 (TGFBR1). Investigation of TGFBR1 has focused on the common genetic variant rs11466445, a short exonic deletion of nine base pairs which results in truncation of a stretch of nine alanine residues to six alanine residues in the gene product. While the six alanine (*6A) allele has been reported to be associated with increased risk of CRC in some population based study groups this association remains the subject of robust debate. To date, reports have been limited to population-based case–control association studies, or case–control studies of CRC families selecting one affected individual per family. No study has yet taken advantage of all the genetic information provided by multiplex CRC families.

**Methods:**

We have tested for an association between rs11466445 and risk of CRC using several family-based statistical tests in a new study group comprising members of non-syndromic high risk CRC families sourced from three familial cancer centres, two in Australia and one in Spain.

**Results:**

We report a finding of a nominally significant result using the pedigree-based association test approach (PBAT; p = 0.028), while other family-based tests were non-significant, but with a p-value <; 0.10 in each instance. These other tests included the Generalised Disequilibrium Test (GDT; p = 0.085), parent of origin GDT Generalised Disequilibrium Test (GDT-PO; p = 0.081) and empirical Family-Based Association Test (FBAT; p = 0.096, additive model). Related-person case–control testing using the “More Powerful” Quasi-Likelihood Score Test did not provide any evidence for association (M_QLS_; p = 0.41).

**Conclusions:**

After conservatively taking into account considerations for multiple hypothesis testing, we find little evidence for an association between the TGFBR1*6A allele and CRC risk in these families. The weak support for an increase in risk in CRC predisposed families is in agreement with recent meta-analyses of case–control studies, which estimate only a modest increase in sporadic CRC risk among 6*A allele carriers.

## Background

Several genome-wide studies [1-3] have provided evidence for significant genetic linkage between a chromosomal region on 9q22 and an increased risk of colorectal cancer (CRC). A further study confirmed this linkage signal and fine-mapped the association to a region centred around 98.15 Mb [4]. Biologically, this chromosomal region houses several interesting candidate CRC susceptibility genes including *PTCH1*, *XPA, GALNT12* and *TGFBR1*[5]. Follow up efforts have particularly focused on *TGFBR1* (hg19 coordinates, chr9:101.87-101.92 Mb), but with largely inconclusive results [3,5-7].

The transforming growth factor β receptor type 1 (*TGFBR1*) gene is an attractive candidate as TGF-β signalling plays an important role in the control of a range of biological functions associated with colon carcinogenesis including tissue homeostasis, angiogenesis, inflammation, proliferation and cellular differentiation and has and has also been implicated in both the suppression and promotion of CRC (see [1] for a recent review). On binding of the TGF-β ligand to TGFBR1, this serine/threonine protein kinase-containing receptor forms a heteromeric complex with type II TGF-β receptors thereby transducing the TGF-β signal from the cell surface to the cytoplasm. A common variant of *TGFBR1*, rs11466445 (heterozygote frequency 0.211; dbSNP135), contains a deletion of three GCG triplets from the sequence of exon 1, resulting in the expression of a mutant receptor protein with six consecutive alanine (TGFBR1*6A) rather than nine consecutive alanine (TGFBR1*9A) residues. This is a hypomorphic mutation encoding a TGFBR1 variant protein with reduced TGF-β growth inhibition-signalling activity. The TGFBR1*6A allele has been proposed to act as a low-penetrance susceptibility allele for a number of malignancies [8], perhaps acting by decreasing *TGFBR1* allelic expression. Allele specific expression (ASE) of TGFBR1 in peripheral blood lymphocytes has been observed, with decreased expression associated with the *6A allele and two other SNPs in linkage disequilibrium [9]. Another study examined SNPs in the 3′ untranslated region of TGFBR1 and found that 29 of 138 patients with MSI-negative CRC showed ASE, with 14 of the 29 (48%) having a *6A/*9A genotype and clear enrichment of ASE in familial cases [10].

Although some studies have suggested that the TGFBR1*6A allele confers an elevated risk of colorectal cancer [5,8,11], most studies have not found such an association [12-17]. A recent large meta-analysis of rs11466445 and colorectal cancer risk assessed nine association studies totalling 6,765 CRC patients and 8,496 unrelated controls and found that heterozygous *6A/*9A carriers showed a significantly increased risk of CRC with a pooled odds ratio (OR) of 1.12 (95% CI = 1.02–1.23; p = 0.013) compared to homozygous *9A/*9A carriers [18]. A further recent meta-analysis, which included 15 subgroups (7,154 case and 8,851 controls), did not find an association with CRC with overall significance (OR = 1.085, 95% CI = 0.963, 1.222; additive model), but instead found a significant association with breast and ovarian cancer. The difference from the previous meta-analysis was the exclusion of one study and the inclusion of two further studies [19]. One of the included studies genotyped rs11466445 in a Spanish cohort somewhat enriched for familial cancer, with ~15% of cases having an affected first-degree relative and found it to be borderline significant with diagnosis of CRC (p = 0.0491; 515 cases, 515 controls) [5]. In the context of familial CRC in particular, two studies have examined families with genetic predisposition [15,20]. In both studies, a case–control design was used - drawing on only one affected member from each family and comparing this group with unrelated controls. In each instance, TGFBR1*6A was not found to be associated with an increased familial colorectal cancer risk. Interestingly, a further study found evidence that the TGFBR1*6A allelic frequency is higher amongst familial CRC patients with mismatch-repair (MMR) negative disease [21].

There have been no reports to date that have explored the likelihood of an association of TGFBR1*6A with hereditary CRC using any family-based association test (FBAT) [22-24], or family-based case–control test designed for related individuals [25]. The family of FBATs examine associations within family groups and so are robust to population stratification, a known confounder of case–control studies [24]. It has been suggested this robustness comes at some cost. Simulations show that classical FBATs are less powerful than case–control tests [24,26], as the latter examine between-family associations instead of exclusively within-family associations. Counter to this argument, the groups of affected relatives sampled from multiplex families should have more power to detect an association due to the higher than expected frequency of susceptibility alleles, compared with affected individuals having sporadic disease [25]. It is also possible to use quasi-likelihood score (QLS) tests, an alternative class of tests to FBATs with different theoretical underpinnings. As opposed to within-family tests, these are between-family case–control tests that can account for the correlation between individuals in families [25].

We recently completed a new genome-wide linkage study [27] using non-syndromic CRC families from three distinct regions in Australia and Spain. One of the linkage regions of interest identified in that study was located on chromosome 9q, proximal to the previously reported 9q22 linkage region, which contains the *TGFBR1* locus. We genotyped an expanded set of families for rs11466445 and used FBATs and the “More Powerful” Quasi-Likelihood Score Test (M_QLS_) to test for association with diagnosis of colorectal neoplasia (i.e. either colorectal adenocarcinoma or advanced adenoma). We report that after applying several family-based association tests we only found a nominally significant result using the PBAT rapid algorithm (p = 0.028), with another three FBAT algorithms all non-significant, but each yielding a p-value <; 0.10. There was no evidence of an association using the M_QLS_ case–control model (p = 0.41).

## Methods

### Ethics statement

The study was reviewed and approved by the Human Research Ethics Committees of the three participating centres: Flinders Medical Centre, Adelaide, The Royal Melbourne Hospital, Melbourne and Institut Català d’Oncologia, Barcelona, with informed consent obtained from all participants.

### Family members

A total of 414 individuals (172 males and 242 females), from 146 CRC families were recruited from clinics in Melbourne, Adelaide and Barcelona and informed consent was obtained from all participants. We restricted our study to non-syndromic high risk CRC families, defined as those containing at least one affected person who has one or more first-degree affected relatives and where the known causal mutations had been excluded. In each case, the diagnosis was confirmed by medical and pathology reports. FAP and MUTYH were excluded clinically and HNPCC or Lynch syndrome was excluded by testing for microsatellite instability (MSI) (as measured by tumour-associated length variation in microsatellites BAT-25 and BAT-26) and/or immunohistochemistry indicating loss of hMLHI, hMSH2, hMSH6 and hPMS2 encoded proteins. Affected status was defined as diagnosis with either colorectal adenocarcinoma (CA) or one or more advanced adenomas (AA), where AA was defined as three or more synchronous or metachronous adenomas and/or adenoma (s) with villous morphology, and/or with severe dysplasia, and/or diameter ≥ 10 mm. Diagnoses were confirmed by pathology reports.

Unaffected individuals were family members who were either over 70 years of age with no history of CA or AA or were 50 years of age or older and had, within the last 5 years, recorded a colonoscopy result negative for neoplasia. As the age of onset is fairly late with a mean age of onset is 55.4 years (Table 1), the cohort is mostly sibships with missing parental genotypes. However, there is inclusion of some extended pedigrees of up to four generations (including non-genotyped founders) containing parent–child, avuncular or cousin pairs. We reclassified 11 young “unaffected” people and those with previous detection of colorectal polyps as “unknown” in accordance with our previous work [27,28]. Of these 11 people, three were heterozygous *6A/*9A genotype, six had the common *9A/*9A genotype and two the rare *6A/*6A genotype. When affecteds are misclassified as unaffecteds, family-based tests that make use of discordant information lose power [29], so it is sensible to reclassify particularly young unaffecteds as having unknown phenotype. All people in the study had their age at blood draw recorded.

**Table 1:** Participant characteristics and demographics

**Participant characteristics**	
**Number of individuals**	
Total including founders	759
Genotyped subjects	414
Flinders centre for cancer prevention and control	202
The Royal Melbourne Hospital	165
Institut Català d’Oncologia	33
Peter MacCallum Cancer Centre	14
**Family structures**	
Number of families	147
Singletons	24
2 generations (nuclear)	87
3+ generations (extended)	36
Trios	25
**Traits of genotyped subjects**	
Total Affected	180
Colorectal adenocarcinoma	145
Advanced adenoma (s)	35
Unaffected relatives	223
Unknown affection	11
Relative Pairs	208
Sib	169
Cousin	1
ParentChild	15
Avuncular	19
Other	4
Female	58.45%
Age, mean +/− SD	56.4 ± 12.6
Affected Age, mean +/− SD	55.4 ± 12.5
Unaffected Age, mean +/− SD	57.2 ± 12.7

### Genotyping

**Figure 1:**
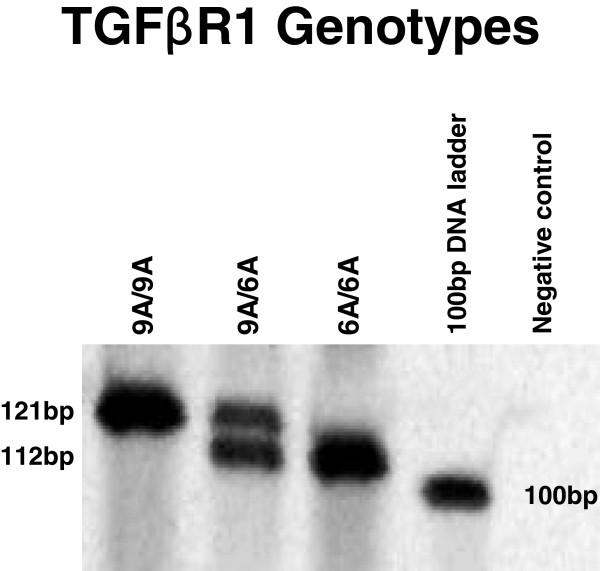
**Genotyping example.** In an electrophoresis gel, the TGFBR1*6A allele migrates as a 112 bp species and the TGFBR1*9A allele migrates as a 121 bp species. Examples of homozygotes and heterozygotes of the two alleles are shown.

The *TGFBR1* rs11466445 variant status was determined by PCR amplification using primers Fwd 5’-GAGGCGAGGTTTGCTGGGGTGAGG-3’ and Rev 5’-CATGTTTGAGAAAGAGCAGGAGCG-3’. PCR amplification was performed in a 25 μL reaction containing 50 ng genomic DNA using the Platinum Taq DNA polymerase with the addition of 3 × enhancer solution and followed the manufacturer’s protocol for GC-rich fragments (Invitrogen). Amplified fragments were separated by electrophoresis on a 10% polyacrylamide gel (Biorad) post-stained with gel-red (Jomar Diagnostics). Genotypes were assigned according to fragment sizes. A product size of 121 bp corresponded to the most common allele, *9A, whereas a product size of 112 bp corresponded to the *6A allele (Figure 1).

### Statistical analyses

Testing for deviation from Hardy-Weinberg equilibrium and Mendelian inconsistencies was performed using Pedstats [30]. The Generalised Disequilibrium Test (GDT) V0.1.1 software [24] was used to test for association in dichotomous relative pairs with identity-by- descent (IBD) statistics estimated using Merlin V1.1.2 [31] Generalised Family-Based Association Tests (FBAT) were undertaken in the FBAT V2.0.4 beta software [22] and PBAT version 3.6 software [23]. FBAT was set to calculate empirical variance estimates and to use a null hypothesis of linkage and no association and p-values were generated from the asymptotic Normal distribution. For PBAT, we used the rapid algorithm, a null hypothesis of linkage and no association with sandwich variance estimation and p-values were generated using an empirical permutation-based method with 10,000 replicates. Sandwich estimation was also used to estimate the correlation between members of larger pedigrees. For time-to-onset analysis, the Wilcoxon Logrank FBAT statistic was examined.

For case–control testing the “More Powerful” Quasi-Likelihood Score Test (M_QLS_) was used [25]. The M_QLS_, an improvement on the quasi-likelihood score test W_QLS_[32], is a case–control test for allelic association that conditions on the pedigree structure using unconditional corrected variance to account for the relatedness amongst individuals. The M_QLS_ can incorporate unaffected controls and controls of unknown affection state. It also makes use of the affection state of relatives with missing genotype data by using their affection status to weight the family. The rationale being that an affected person who has additional affected relatives is more likely to be carrying a genetic predisposition.

### Accounting for linkage

The use of null hypotheses of “linkage and no association” in the FBAT and PBAT software was conservative. While the families show genetic linkage with cancer diagnosis in a region of chromosome 9 (9q33.3–9q34.3; non-parametric LOD = 2.24) with a 1-LOD support interval of ~127.97–140.0 Mb [27], this does not cover the location of the *TGFBR1* locus at 101.9 Mb and this region is only weakly linked with CRC. At the SNP rs928180, which is in the *TGFBR1* intragenic region, the non-parametric (S_*all*_) LOD score is 0.293. By using IBD information it is possible to control for linkage using the GDT. Unlike the FBATs, the M_QLS_ case–control test does not control for linkage and allows both linkage and association to contribute to the test statistic.

## Results

### Genotyping and quality control

We found 315, 95 and four people to be homozygous for the rs11466445 *9A allele, heterozygous and homozygous for the *6A allele, respectively, with a *6A allele frequency (AF) of 0.124. The four people carrying the *6A/*6A genotype were dispersed across two families, each having one discordant pair (one affected and one unaffected individual). An exact test found the genotype to be in Hardy-Weinberg equilibrium (all individuals, p = 0.3687; 126 unrelated individuals, p = 1.0) and there were no observed Mendelian inconsistencies. The allele frequencies of the *6A allele in the affected and unaffected family members (affected family member, AF = 0.117, unaffected family member, AF = 0.130) were slightly higher than observed in a case–control British study of hereditary CRC (913 cases, AF = 0.096; 828 controls, AF = 0.100) [15] and a further Swedish Caucasian cohort with hereditary non-polyposis colorectal cancer (HNPCC) and non-HNPCC hereditary CRC patients (83 HNPCC + 179 non-HNPCC cases, AF = 0.107; controls, AF = 0.106) [20].

### Family-based association testing

In the first instance, we tested for an association with colorectal neoplasia using the Generalised Disequilibrium Test (GDT). Given the large differences in pedigree sizes in this present study and the high number of possible intra-pedigree discordant pairings between genotyped people, the generalised relative pairs weighted by family size approach implemented by the GDT software, provides a good fit with the data. One caveat of using discordant pairs, however, is that in complex disease some people inheriting a risk allele do not develop the disease, or develop it rather late in life and this needs to be taken into account. As some of the pedigrees are multi-generational, we used inheritance by descent (IBD) data to inform the GDT analysis. Testing for association between the rs11466445 *6A allele and colorectal neoplasia in 208 discordant relative pairs by the GDT algorithm produces a p-value of 0.085 (Table 2). Inclusion of gender as a covariate did not change the p-value. While this result is not significant at a 5% level, given the borderline p-value and to avoid false negative results, we further tested the association using other family-based association methods that construct a test with different assumptions and/or make use of different groupings of related people within the data. For this, we ran a parent of origin GDT test and also the tests implemented in the FBAT and PBAT software.

**Table 2:** Association results

**Model**	**Allele**	**Allele Freq**	**Informative units**	P-value*	**Statistic**
**GDT**			*Relative Pairs*		
Allelic	6	0.124	208	0.085	1.724
**Parent of origin GDT**			*Parent–child pairs*		
Allelic	6	0.124	15	0.081	1.746
**FBAT (empirical)**			*Nuclear families*		
Additive	6	0.115	22	0.096	1.665
Dominant	6	0.115	23	0.193	1.301
**PBAT (rapid)**			*Trios*		
Additive	6	0.114	26	**0.028**	-
**MQLS**			*Cases/Controls*		
Allelic	6	0.124	180/137	0.41	0.82

*- All p-values rounded to 3 decimal places; nominally significant p-value is in bold.

The GDT software allows analysis to be constrained to only examine discordant parent–child pairs (GDT-PO) and ignore unaffected sibling data. This parent of origin test for the 15 parent–child pairs in the study was consistent with the full GDT result (p = 0.081; Table 2). Next, we tested the association using the Family-based association test (FBAT), a statistic that examines the covariance between phenotype and allele transmission (Mendelian residuals) from parents to offspring. Considering there are only four homogyzous *6A carriers we did not test the recessive genetic model. As the variant falls in an area of weak genetic linkage, FBAT empirical variance estimates were used to control for correlation amongst sibling genotypes within pedigrees. The FBAT result was non-significant (Table 2). Given the large number of missing parents in the current study and only having 22 (additive model) or 23 (dominant model) informative nuclear families, there is some reliance upon the sufficient statistic and large sample theory. Regardless, the p-value for the *6A allele under an additive model (p = 0.096) is close to that obtained with the GDT (p = 0.081), which uses a robust measure not dependent upon large sample theory.

Finally, we tested for an association under an additive model with the *6A allele using the FBAT implemented in the PBAT software. Using the PBAT rapid algorithm, the association was found to be nominally significant under an additive model (p = 0.0278; 10,000 permutations) with a null hypothesis of linkage and no association, with robust sandwich variance estimates (Table 2). We also tested for an association between the *6A allele and age of CRC diagnosis, but found no evidence (additive model, FBAT-Wilcoxon, p = 0.150, null hypothesis – linkage, no association with sandwich variance).

### Case–control testing

We used the “More Powerful” Quasi-Likelihood Score Test (M_QLS_) which accounts for relatedness between subjects using a corrected variance. Unlike the FBATs, the M_QLS_ can make use of the genotyping information of the 24 singletons in the study and can use the people with unknown affection status as controls. The result was insignificant (Table 2), with a p-value of 0.41 (180 cases, 137 controls) and specifying a disease prevalence of 0.05. The result was highly insensitive to specifying other disease prevalence values and setting prevalence to 0.001, 0.1 and 0.2 gave p-values of 0.39, 0.41 and 0.44, respectively. Unlike FBATs, the M_QLS_ is not robust to population heterogeneity and will inflate type I error rates (the incorrect rejection of a true null hypothesis) in instances of population stratification. Given the convincingly non-significant result we did not investigate this further.

## Discussion

Testing with the rapid PBAT algorithm gave a nominally significant result under an additive genetic model (p = 0.028). Under the GDT, GDT-PO and FBAT approaches we did not find a significant association, but all the p-values were consistently borderline, with p-values <; 0.10. Unlike the FBAT approaches, we found no evidence of an association using M_QLS_, a case–control method that corrects for relatedness amongst subjects (p = 0.41).

The differences in p-values between the methods, under the same hypotheses and genetic models are due to the formulation of the test and also the treatment of family structures, which leads to differences in groupings informative for the test statistic.

The GDT, a robust generalisation of the intuitively simple transmission-disequilibrium test (TDT), examines transmission disequilibrium between pairs of discordant relatives. The variant GDT-PO test, considers only parent–child discordant pairs. As relatively few parent–child pairs were genotyped in this study, the test will have much reduced power. However, given the age of the parents, the result should be more robust to misspecification of phenotype. The FBAT and PBAT algorithms are highly related and examine transmission disequilibrium from parents to affected offspring.

For the FBAT statistic, informative families are those with at least one parent heterozygous for the two *TGFBR1* alleles and having affected offspring. The use of only affected offspring in the association statistic makes it robust to phenotype misspecification of affected people as unaffected. In the case of a missing parent, or parents, the test conditions on the sufficient statistic for the genotype distribution in each family; where a parent genotype is expressed as a set of likelihoods conditioned upon known offspring genotype(s). The design of the FBAT necessitates that extended pedigrees are split into nuclear families, which can introduce bias due to correlation. The FBAT also requires specification of the genetic model. The PBAT rapid algorithm differs from FBAT in that extended pedigrees are broken up into clusters of trios who share the same parents. The rapid algorithm in PBAT tests only the minor alleles and offers the ability to generate p-values using a robust Monte Carlo permutation based method instead of the asymptotic Normal distribution [23]. It also offers time to onset analyses with the same empirical p-values. Finally, the M_QLS_ test is very different to the others, and is a regression model rather than a family-based association test. It considers both within- and between-family associations using a linear regression of genotypes on affection status with correlations for relatedness modelled as a kinship coefficient random effect.

The closeness in p-values between all the family-based methods demonstrates the finding is not particularly sensitive to different assumptions underpinning these various algorithms. More broadly, given the difference in informative pairs/families in each FBAT method and nature of the algorithm, the general agreement across methods suggests this marginal evidence of an association is not a spurious result.

However, the nominally significant PBAT result should perhaps be treated with some caution. While the p-value was generated using a robust empirical Monte-Carlo based method, it is possible the partitioning of people into clusters of trios may inflate type I error. As the 22 informative nuclear families are broken into 26 informative clusters of trios, there is a degree of correlation between some clusters that is unaccounted for by the approach. Conversely, there is reason to think that such correlation may not greatly influence the p-value. The similarity in p-values between the GDT and the FBAT, which also splits extended pedigrees, suggests the difference in handling of extended pedigree structure between the methods did not overly affect the association test result in this instance.

In essence, a method (PBAT) which examines transmission to affected relatives but breaks pedigrees up for computational reasons is significant, while a method (GDT) that examines discordant relative pairs that does not adjust pedigree structure is non-significant. It is unclear how the different pairings or pedigree structure between these methods is contributing to the difference in p-value. In simulations, the GDT test was found to have more power over the FBAT in the majority of nuclear family and extended pedigree structures tested [24]. However, nuclear families with two missing parents (which include most of this present cohort) were not simulated, so it is possible the FBAT implemented in the PBAT software is more powerful in this scenario and may help explain the lower p-value.

Only one SNP was tested for association with CRC, however, the genotype data was reformulated into several tests and genetic models with different treatment of the genotype data and family structures. Correction for these multiple tests can be applied, but such correction assumes independence of the tests. As these tests are very dependent, such a correction is highly conservative. Given the number of tests made of this single hypothesis, the PBAT association will become non-significant after correction for multiple tests.

All reported association studies of rs11466445 have been of a case–control design [18]. Even studies that have gathered affected cases from families with an inherited predisposition have used a case–control design, with one case selected from each family and compared to unrelated controls [15,20]. To our knowledge, this present study is the first to examine the association between rs11466445 and colorectal cancer using family-based association statistics or case–control methods for related individuals. Our study is designed to examine the association of rs11466445 with colorectal neoplasia diagnosis within families predisposed to CRC. The within-family approach frees the analysis from concerns about population stratification.

Collectively, current evidence suggests the *6A allele is a relatively minor contributor to CRC prevalence. A modest 12% and 8.5% increase in CRC risk, respectively, was found by two meta-analyses across large populations [18,19]. The first meta-analysis found the *TGFBR1* *6A allele to be significantly associated with CRC while the latter did not. The authors of a recent review considering the *6A allele association and ASE studies together concluded that the effect of the allele on CRC predisposition is, at best, very subtle [33].

## Conclusions

Our finding here of little evidence for an association with 6*A in CRC predisposed families supports the conclusions of recent meta-analyses and a review, which find the effect of the 6*A allele on CRC risk is modest. This weak evidence for association, together with the modest linkage signal in the region of the *TGFBR1* locus, suggests that rs11466445 does not contribute significantly to the collective genetic predisposition towards CRC in these families.

## Abbreviations

6*A: Six alanine allele; AA: Advanced adenoma; ASE: Allele specific expression; CA: Colorectal adenocarcinoma; CRC: Colorectal cancer; FBAT: Family-based association test; GDT: Generalised disequilibrium test; GDT-PO: Parent of origin generalised disequilibrium test; HNPCC: Hereditary non-polyposis colorectal cancer; IBD: Identity-by- descent; M_QLS_: “More Powerful” quasi-likelihood score test; MSI: Microsatellite instability; PBAT: Pedigree-based association test; QLS: Quasi-likelihood score.

## Competing interests

The authors declare that they have no competing interests.

## Authors’ contributions

LJL performed all the genotyping and experimental lab work, prepared data for analysis and helped with the manuscript; GSB performed experimental lab work; JPR, BT and IWS analysed the data; GPY, FM, IB and GC coordinated the original collection of the samples. JPR, BT and GNH wrote the manuscript; TJL contributed programme support and insightful critique; GNH conceived and designed the current study. All authors participated in data interpretation and critical revision of the manuscript. All authors read and approved the final manuscript.

## Pre-publication history

The pre-publication history for this paper can be accessed here:

http://www.biomedcentral.com/1471-2407/14/475/prepub
